# Associations between pain intensity, psychosocial factors, and pain-related disability in 4285 patients with chronic pain

**DOI:** 10.1038/s41598-024-64059-8

**Published:** 2024-06-12

**Authors:** Live Landmark, Hans Fredrik Sunde, Egil A. Fors, Leif Edward Ottesen Kennair, Annahita Sayadian, Caroline Backelin, Silje Endresen Reme

**Affiliations:** 1 Department of Psychology, Faculty of Social and Educational Sciences, Norwegian University of Science and Technology, Trondheim, Norway; 2 Department of Public Health and Nursing, Faculty of Medicine and Health Sciences, Norwegian University of Science and Technology, Trondheim, Norway; 3 Department of Psychology, Faculty of Social Sciences, University of Oslo, Oslo, Norway; 4 Centre for Fertility and Health, Norwegian Institute of Public Health, Oslo, Norway; 5 Department of Pain Management and Research, Oslo University Hospital, Oslo, Norway

**Keywords:** Psychology, Human behaviour

## Abstract

Pain, a widespread challenge affecting daily life, is closely linked with psychological and social factors. While pain clearly influences daily function in those affected, the complete extent of its impact is not fully understood. Given the close connection between pain and psychosocial factors, a deeper exploration of these aspects is needed. In this study, we aim to examine the associations between psychosocial factors, pain intensity, and pain-related disability among patients with chronic pain. We used data on 4285 patients from the Oslo University Hospital Pain Registry, and investigated pain-related disability, pain intensity, pain catastrophizing, psychological distress, perceived injustice, insomnia, fatigue, and self-efficacy. We found significant associations between all psychosocial variables and pain-related disability, even after adjusting for demographic factors. In the multiple regression model, sleep problems and pain intensity were identified as primary contributors, alongside psychological distress, and fatigue. Combined, these factors accounted for 26.5% of the variability in pain-related disability, with insomnia and pain intensity exhibiting the strongest associations. While the direction of causation remains unclear, our findings emphasize the potential of interventions aimed at targeting psychosocial factors. Considering the strong link between psychosocial factors and pain-related disability, interventions targeting these factors—particularly insomnia—could reduce disability and enhance quality of life in those who suffer.

## Introduction

Pain is a common complaint that affects a substantial portion of the population^1^. In Norway chronic pain touches the lives of approximately one in three adults^2^. Unlike short-lived acute pain, which tends to fade as the underlying cause resolves, chronic pain is characterized by its persistence, often transitioning into a long term condition after the first year^3^. In addition to pain duration and other temporal aspects, the International Association for the Study of Pain (IASP) suggest location, pain quality and pain intensity as the major “axes of pain”^4^. Chronic pain can be further classified into two categories in the recent ICD-11 taxonomy: primary pain, which lacks clear underlying causes, and secondary pain, which emerges as a symptom of an underlying disease^5^. Both forms of chronic pain play a significant role in prolonged sick leave and work-related disability^6^.

Pain, defined as an “unpleasant sensory and emotional experience associated with actual or potential tissue damage”^7^, is influenced not only by physical sensations but also by psychological and social factors^8^. Since purely biomedical treatments are limited in their effectiveness for chronic pain, the understanding of psychological and social dimensions becomes increasingly important. People living with chronic pain not only contend with the burden of physical symptoms but also face its impact on daily functioning, commonly referred to as *pain-related disability*^9^. To refine treatments and prevent pain-related disability in individuals with chronic pain, we need to comprehensively explore psychosocial aspects^10^ in addition to the pain axes.

The association between pain intensity and the impact on functional abilities, referred to as pain-related disability, remains uncertain due to the inconsistent findings in existing research^11^. This uncertainty suggests that factors beyond pain intensity, such as widespread pain and psychosocial co-morbidity, significantly contribute to pain-related disability^12,13^. Studies have indicated that cognitive behavioral therapy (CBT) can reduce disability by diminishing maladaptive responses like pain-related fear and catastrophizing, while strengthening self-efficacy. However, the effectiveness of CBT in reducing pain intensity does not appear to outperform that of control treatments^14^. Consequently, further research is essential to gain a better understanding of the factors influencing pain-related disability.

Various models have been developed to understand the interplay between pain experience and behaviour, with the fear-avoidance model being one of the most established^15^. According to this model, individuals in pain may develop excessive fear and avoidance behaviors due to their perceptions and beliefs about pain. This fear and avoidance contribute to the exacerbation and perpetuation of pain-related disability^16^. While fear can serve as an adaptive response to acute pain, excessive focus on pain and catastrophic thinking can result in maladaptive cognitive and behavioural responses^17^. Pain catastrophizing, characterized by negative rumination, plays a crucial role in predicting chronic pain^18^. It triggers unconscious fear and fosters pain-avoidant behaviours^19^. The model underscores the importance of addressing and breaking this destructive cycle of fear and avoidance for improving pain-related outcomes. Avoidance of pain-triggering situations predicts pain-related disability and is often accompanied by negative expectations and emotions^20^. Individuals who engage in extensive pain catastrophizing tend to experience higher levels of pain intensity in the short term but are also at a greater risk of developing chronic pain and pain-related disability in the long term^18,21^.

Psychological distress is recognized as a risk factor for the development of chronic pain^22^, and persistent psychological distress and lifetime stressors are particularly implicated in the progression of chronic pain^23,24^. Additionally, pain-related injustice, characterized by negative appraisals of pain-related losses and unfairness, has consequences for prognoses and treatment outcomes^25^. It fosters hypervigilance toward pain and exacerbates the pain experience^26^. The impact of various forms of psychological distress can vary and influence the extent and duration of disability^27,28^.

Psychological distress can also initiate and perpetuate physiological stress activation. The Cognitive Activation Theory of Stress (CATS) theory offers insights into how stress impacts health^29^. According to CATS, individuals´ perceptions and interpretations of perceived stressors are crucial. When individuals feel helpless or hopeless, the stress responses will persist, potentially leading to illness or disease. Furthermore, a bidirectional relationship exists between chronic stress and sleep disturbances, particularly insomnia, which is prevalent in chronic pain patients^30^ and often results in fatigue^31^, a common complaint among individuals with chronic pain^32^. Recent studies have also revealed the predictive value of sleep disturbances and fatigue^33,34^, warranting further investigation in diverse pain populations.

On the other hand, self-efficacy, defined as an individual’s belief in their ability to perform tasks or achieve goals^35^, appears to serve as a buffer against heightened stress levels. This belief enhances motivation and shapes the pain experience by fostering confidence in one’s ability to influence their suffering^35^. Improved pain-related self-efficacy is associated with enhanced coping abilities^36^. Furthermore, resilience plays a vital role in buffering against stressors that could impact the pain experience.

Studies have consistently linked the mentioned factors to adverse outcomes for people with pain, except for self-efficacy, which tends to have the opposite effect and be beneficial^19,25,28,37^. A recent meta-analysis found for instance support for the various components of the fear-avoidance model in patients with chronic pain, where fear of pain, pain catastrophizing, and pain vigilance, were all strongly associated with negative affect, anxiety, pain intensity, and disability^38^. Similar findings were reported in a systematic review focused on studies of musculoskeletal pain, which identified an association between increased pain-related fear and anxiety and higher levels of pain intensity and disability^39^. Alamam et al.^40^ extended this research to show that across cultures and regions, beliefs about pain, including self-efficacy, pain catastrophizing, and pain-related fear, were associated with disability due to low back pain^40^. Other studies have focused more specifically on the association between pain and disability. Here, self-efficacy, psychological distress, and fear were identified as mediators in the relationship between pain and disability, although catastrophizing did not show a mediating effect in this meta-analysis^41^.

Despite these insights, it is important to note that most current studies have focused on musculoskeletal pain, particularly back pain. There is as such a need to investigate how these associations hold in more general pain populations to better understand the broader implications of psychosocial factors on pain-related disability.

Building on previous studies, we hypothesize that various psychosocial factors—such as pain catastrophizing, psychological distress, perceived injustice, insomnia, fatigue, and self-efficacy—are linked to pain-related disability in patients with chronic pain. In our study, we seek to enhance the existing literature by confirming the already established significant relationship in a large, real world Norwegian patient cohort of patients with diverse chronic pain conditions referred to a multidisciplinary pain clinic. Additionally, as an exploratory analysis, we aim to assess how indicators of chronicity, such as work status and duration of pain, moderate these relationships^42^. To investigate these relationships, we have formulated three research questions:

1. How do various psychosocial factors correlate with pain-related disability in chronic pain patients?
2. Do these relationships differ depending on work disability status (temporary vs permanent financial disability benefits) or pain duration (< 1 vs ≥ 1 year)?
3. What proportion of the variance in pain-related disability can be explained by these psychosocial factors collectively?

## Methods

### Sample

We utilized questionnaire data from the Oslo University Hospital Pain Registry, which includes information from all patients dealing with chronic pain and attending the Oslo University Hospital Pain Registry, totaling 4285 patients. This data was collected between October 2015 and March 2020^43^. The patients were referred to the registry from general practitioners or specialist health care services and received treatment within the South-Eastern Health region of Norway, encompassing Oslo, the country’s largest city. The data collection occurred in a naturalistic clinical audit setting. Some patients were given the option to choose between a comprehensive questionnaire package and a shorter version, which included only a few selected scales to reduce the potential survey burden.

This patient population was notably diverse, representing a range of chronic pain conditions, including those related to cancer, heart disease, multiple sclerosis, chronic obstructive pulmonary disease, and various subacute pain conditions. These conditions encompassed cases with and without a known explicit cause. It’s worth noting that non-malignant cases were overrepresented^44^. Another study conducted on the same cohort (n = 2799) reveals that patients with primary pain constitute more than half of the patient population^45^. The overview is as follows: Chronic primary pain 52.5% (n = 1469), Chronic cancer-related pain 0.3% (n = 8), Chronic postsurgical or posttraumatic pain 6.4% (n = 178), Chronic secondary musculoskeletal pain 12% (n = 335), Chronic secondary visceral pain 1.4% (n = 38), Chronic neuropathic pain 16.3% (n = 456), Chronic secondary headache or orofacial pain 0.3% (n = 8) and Unspecified chronic pain 11% (n = 307).

### Measures

The patients supplied demographic information, including their age (in years), gender (male/female), level of education (primary, secondary, short tertiary, and long tertiary), employment status (working vs non-working), length of time spent out of work (in years), degree of partial or full financial benefits (Group 1: “Sick pay” up to 1 year and “Work assessment allowance/AAP” up to 4 years or Group 2: “Permanent benefit”). Additionally, they reported the duration of their pain. Since research suggest that neural networks undergo significant changes within the initial year of experiencing pain, and thereafter stabilizing^3^, pain duration was recoded into less than a year versus one year or more^43^.

#### Independent variables

##### Pain intensity and bothersomeness: Numeric Rating Scale (NRS)

The NRS questionnaire consists of two items assessing pain intensity and pain bothersomeness. For instance, it evaluates the pain experienced last week in terms of its highest, average, and lowest intensity, as well as the current pain level^46,47^. Pain intensity and pain bothersomeness are significantly correlated (r = 0.698, n = 3836, p ≤ 0.001), yet they measure different aspects of pain, representing two separate dimensions of the pain experience and therefore both are included. Each question offers 11 response options, spanning from 0 (“No pain at all”) to 10 (“Worst pain possible”)^46^. Mild pain intensity typically ranges from 1 to 3, moderate pain intensity ranges from 4 to 6, while scores of 7 and above are considered severe pain intensity^48^. There are no established cutoff scores for bothersomeness. The NRS has been established as a reliable and valid measure^49^. The two pain intensity and bothersomeness measures are based on the well-validated Brief Pain Inventory^50^, which has also been validated in Norwegian^51^.

##### Catastrophizing: Pain Catastrophizing Scale (PCS)

The PCS is a 13-item scale that measures pain catastrophizing, meaning “an exaggerated negative mental set brought to bear during actual or anticipated painful experience”^52^. Each question offers five response options, ranging from “Not at all” (0) to “All the time” (4), with a higher score indicating more intense pain catastrophizing. The scores range from 0 to 52, with individuals scoring 24 points or more being considered catastrophizers and those scoring below 15 points being considered non-catastrophizer^52^. The PCS has demonstrated reliability and validity, including a Norwegian validation^53^. In this cohort, the Cronbach’s alpha was 0.95.

##### Psychological distress: Hopkins Symptom Check List-25 (HSCL-25)

The HSCL-25 is a 25-item questionnaire that evaluates psychological distress encompassing anxiety, depression and somatization^54^. Each question provides four different response options, ranging from 1 (“Not at all”) to 4 (“Extremely”), with higher total scores indicating greater psychological distress. An HSCL-25 mean score of 1.75 or above suggests the need for treatment^55^. The HSCL-25 is established as a reliable and valid tool and has been validated in Norwegian^55^. In this cohort, the Cronbach’s alpha was 0.94.

##### Perceived injustice: Injustice Experience Questionnaire (IEQ)

The IEQ is a 12-item questionnaire that measures the degree to which patients with chronic pain perceive injustice about their pain condition. It presents respondents with statements reflecting notions of blame or unfairness, as well as the severity and perceived irreparability of the associated losses^56^. Each question has five response options ranging from “Never” (0) to “All the time” (4)^56^. The scores range from 0 to 48, with scores above 19 (medium IEQ) predicting both increased pain severity and more work-related disability, and a cutoff score of 30 (high IEQ) being considered clinically relevant^25^. The IEQ has exhibited reliability and validity in previous studies^57^ and has been validated in Norwegian^58^. Cronbach’s alpha was 0.92 in this cohort.

##### Insomnia: Insomnia Severity Index (ISI)

The ISI is a 7-item questionnaire that assesses insomnia symptoms over the past couple of weeks^59^. This comprehensively examines the nature, severity, and impact of insomnia symptoms, providing insights into the individual’s sleep disturbances and their consequences. Each question has five response options, spanning from 0 (“None”/“Very happy”) to 4 (“Very/Very unhappy”), with higher scores indicating more severe insomnia symptoms. The scores range from 0 to 28, with scores at or above 15 being considered moderate (15–21) to severe (22–28) insomnia^59^. The ISI is known to have high reliability and validity^59^ and has been validated in Norwegian^60^. In this cohort, Cronbach’s alpha was 0.91.

##### Fatigue: Chalder Fatigue Questionnaire (CFQ)

The CFQ is an 11-item questionnaire that measures fatigue, encompassing both physical and mental aspects^61^. Each question offers four response options, ranging from “Less than usual” (0) to “Much more than usual” (3). Following Chalder’s procedure, each item was dichotomized so that respondents answering 2 or 3 were considered “cases” for that particular question, and those answering 0 or 1 were considered non-cases. The total score ranges from 0 to 11, with scores of 4 or above indicating severe fatigue^62^. The CFQ has been established as a reliable and valid measure^61^ and has been validated in Norwegian^63^. In this cohort, Cronbach’s alpha was 0.87.

##### Self-efficacy: General Self-Efficacy scale (GSE)

The GSE is a 10-item questionnaire that measures perceived self-efficacy, regardless of morbidity and disability^64^. Each question has four response options, ranging from 1 (“Not at all true”) to 4 (“Exactly true”), with higher scores indicating greater perceived self-efficacy^64^. The scores range from 0 to 4, and is usually found to average 2.9 across various samples^65^. The GSE has been validated as reliable and valid and has been validated in Norwegian^66^. In this cohort, the Cronbach’s alpha was 0.91.

#### Dependent variable: pain-related disability, Oswestry Disability Index (ODI)

The ODI is a 10-item questionnaire that measures pain-related disability across ten dimensions of daily functioning: pain intensity, personal care, lifting, walking, sitting, standing, sleeping, sexual activity, social engagement, and travel^9^. The Oslo University Hospital Pain Registry employs a modified version of the questionnaire by omitting the word “back” from the question in the introduction^43^. Each question offers six different response options, ranging from 0 to 5, with higher scores indicating more disability. The score is rescaled to range from 0 to 100%. Scores between 0 and 20% indicate minimal disability, scores between 21 and 40% indicate moderate disability, scores between 41 and 60% indicate severe disability, scores between 61 and 80% indicate being crippled, and finally, scores between 81 and 100% indicate either being bedbound or exaggerating symptoms^9^. The ODI is established as a reliable and valid tool^9^ and has been validated in the original version in Norwegian^67^. The Cronbach’s alpha was 0.86 in this questionnaire.

#### Missing data

For each scale, we calculated the mean score for each participant. Participants who responded to less than 50% of the questions were considered as non-respondents. Afterwards, we rescaled all the scales to their original range. For each regression model, we used listwise deletion.

### Ethical considerations

The present study employs a dataset from a comprehensive patient registry, and the specific data provided to us were fully anonymized. Research protocols involving anonymized data in Norway are exempt from the requirement of formal review and approval by an Institutional Review Board (IRB). Complying with these regulations, our project did not necessitate a formal ethics review by the Regional Ethical Committee. Despite this exemption, we have nevertheless conducted our research with a deep commitment to ethical practices. We have implemented rigorous internal ethical reviews to ensure the highest standards of integrity throughout our research. This included verifying that all data used were collected from participants who had given explicit consent for their utilization in research, thereby upholding their rights and autonomy. Moreover, we have taken extensive measures to maintain the security and confidential handling of the research data. Throughout this process, and into the dissemination of our findings, we are dedicated to the ethical principle of honesty and clarity, ensuring that our research is communicated accurately and transparently.

### Statistical analysis

We conducted the analysis using SPSS version 27 and reported descriptive statistics, including frequencies, means, and standard deviations (Tables 1, 2). Figures was made in R using the ggplot function.

To explore the relationship between psychosocial indicators and pain-related disability, we calculated both bivariate and adjusted regression coefficients, with pain-related disability as the dependent variable. Standardized regression coefficients are presented in the figures, while unstandardized coefficients can be found in Table 3 (for unadjusted coefficients, please see Table 3—Supplementary Table S1 provides additional details). Our adjustments included controlling for age, gender, education level (treated as a continuous), and work status (for adjusted coefficients, consult Table 3—see also Supplementary Table S2).

To investigate whether the associations between psychosocial variables and pain-related disability were influenced by financial disability benefit status or pain duration, we used hierarchical regression analyses with interaction terms in separate models, while maintaining control for age, gender, education level, and work. In step 1, we standardized all variables, both independent and dependent, except for the moderators “financial benefit” and “pain duration”, as they are categorical variables. In step 2, we created interaction terms/product terms. In step 3, we conducted a linear regression. We carried out this process as two models. The first model included the control variables. The second model included one interaction term at a time. First, we examined financial disability benefit status, categorizing it into two groups: temporary financial disability benefits and permanent financial disability benefits (see Supplementary Table S3). Individuals who did not receive either temporary or permanent benefits were excluded from this model. Second, we assessed pain duration, categorizing it into two groups: short-term pain-duration (< 1 year) and long-term pain duration (≥ 1 year) (see Supplementary Table S4).

Finally, we evaluated the unique contributions of the included psychosocial measures in explaining the variance in pain-related disability scores. We accomplished this by including all variables in a single regression model, along with the covariates (Table 4), while also checking for multicollinearity.

#### AI-assisted technologies in the writing process

During the preparation of this work the authors used ChatGPT in order to enhance language and readability. After using this tool, the authors reviewed and edited the content as needed and take full responsibility for the content of the publication.

## Results

### Descriptive statistics

The sample consisted of 4285 participants, with women accounting for the majority at 58.1% (n = 2490). The average age of the participants was 49.5 years (SD = 15.59, n = 4285). Approximately 39% (n = 1673) had completed college or university education. A substantial portion, 60.5% (n = 2592), were currently unemployed, while 46.3% (n = 1985) having been out of work for more than two years. Among those not employed, the average duration of unemployment was 10.8 years (SD = 9.72, n = 1847). For the employed participants, the average Full-Time Equivalent percentage was 78.5% (n = 1251).

Around one-third of the participants (36.3%, n = 1553) received temporary financial disability benefits, and another one-third (27.8%, n = 1190) received permanent financial disability benefit. A large majority had experienced pain for one year or more (88.1%, n = 3776). A summary of these sample characteristics is available in Table 1.

**Table 1:** Demographics (N = 4285).

Categorical variables	n	%
Gender		
Men	1689	39.4
Women	2490	58.1
Missing	106	2.5
Education level		
Elementary school (1–10 years)	643	15.0
High school (11–13 years)	1847	43.1
College or University (14–17 years)	1303	30.4
Higher University level (> 17 years)	370	8.6
Missing	122	2.8
Work or life situation		
Working/student/military service	1496	34.9
Not working	2592	60.5
Missing	197	4.6
Out of work		
< 2 year	484	11.3
> 2 year	1985	46.3
Missing	1816	42.4
Do you receive any of the following social welfare benefits?		
Old-age pension, early retirement pension, or survivors’ pension	544	12.7
Sick pay full	256	6.0
Sickpay part	167	3.9
Work assessment allowance (AAP)	1130	26.4
Disability pension, full	1016	23.7
Disability pension, part	174	4.1
Day benefits during unemployment	25	0.6
Social welfare	72	1.7
Other	140	3.3
Missing	761	17.8
Duration of pain		
< 1 year	509	11.9
≥ 1 year	3776	88.1

Continuous variables	n	M (SD)
Age at completion	4285	49.52 (15.59)
Paid work, employment percentage	1251	78.49 (29.98)
Years out of work	1847	10.84 (9.72)

**Figure 1:**
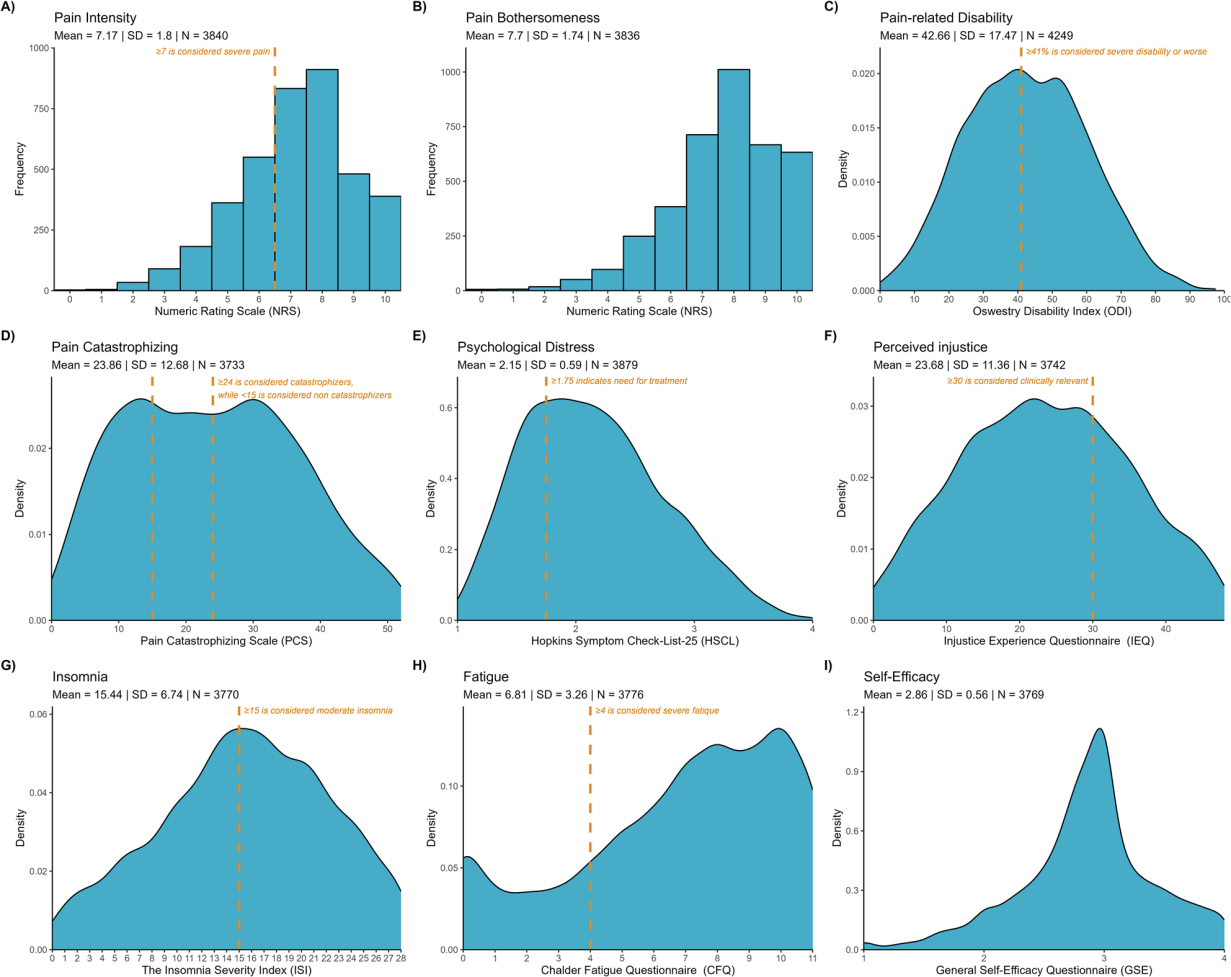
Distributions of variables along with clinically relevant cutoffs.

In terms of pain-related measures, mean pain intensity (M 7.17, SD 1.80, n = 3840) and pain bothersomeness (M 7.70, SD 1.74, n = 3836) exceeded clinically relevant thresholds. Pain-related disability, on average, was severe (42.7%, SD 17.47, n = 4249). Most participants reported levels of pain catastrophizing (M 23.86, SD 12.68, n = 3733), psychological distress (M 2.15, SD 0.59, n = 3879), and perceived injustice (M 23.68, SD 11.36, n = 3742) that indicated a need for treatment. Average insomnia levels were moderate (M 15.44, SD 6.74, n = 3770), with 51.4% reporting clinically relevant insomnia (i.e., score > 15). Similarly, the average level of fatigue was 6.81 (SD 3.26, n = 3776). On the other hand, self-efficacy was within the normal range compared to samples from the general population (M 2.86, SD 0.56, n = 3769). Descriptive statistics for these psychosocial factors are visualized in Fig. 1. Further details are provided in Table 2.

**Table 2:** Clinical characteristics.

	N	Mean	SD
Pain intensity and bothersomeness			
Pain intensity (NRS, range 0–10)	3840	7.17	(1.80)
Pain bothersomeness (NRS, range 0–10)	3836	7.70	(1.74)
Pain-related disability			
Oswestry Disability Index (ODI, range 0–100)	4249	42.66	(17.47)
Pain catastrophizing			
Pain Catastrophizing Scale (PCS, range 0–52)	3733	23.86	(12.68)
Psychological distress			
Hopkins Symptom Check-List-25 (HSCL-25, range 1–4)	3879	2.15	(0.59)
Perceived injustice			
Injustice Experience Questionnaire (IEQ, range 0–48)	3742	23.68	(11.36)
Sleep			
Insomnia Severity Index (ISI, range 0–28)	3770	15.44	(6.74)
Fatigue			
Chalder Fatigue Questionnaire (CFQ, range 0–11)	3776	6.81	(3.26)
Self-efficacy			
General Self-Efficacy scale (GSE, range 1–4)	3769	2.86	(0.56)

### Analyses

**Figure 2:**
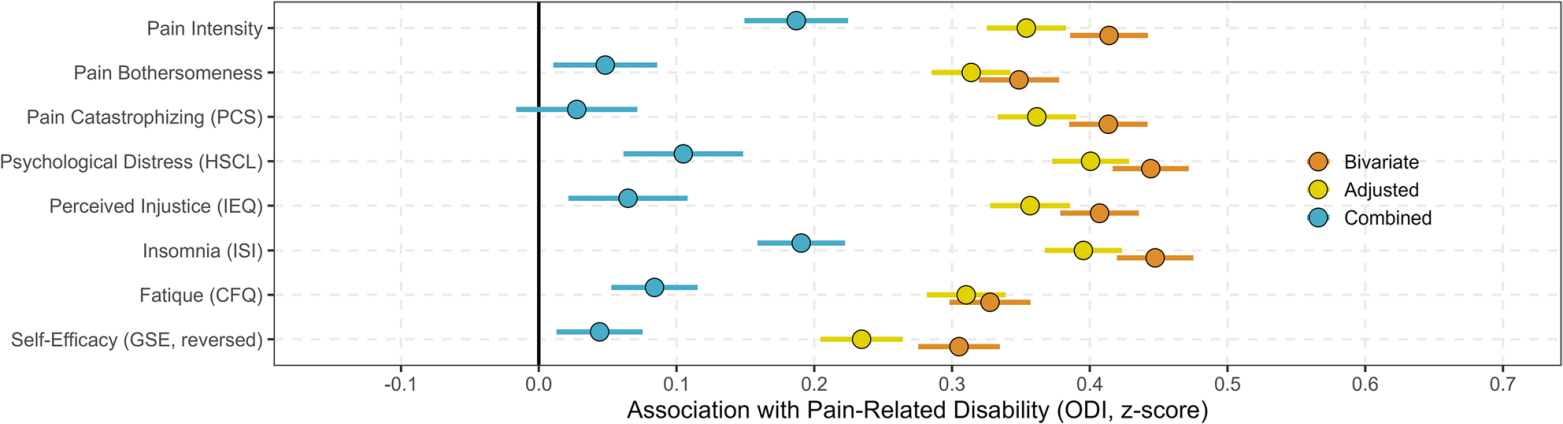
Associations between psychosocial variables and pain-related disability (orange), adjusted for age, gender, education level and work (yellow) and all the psychosocial variables in a combined model including covariates (blue).

We identified strong associations in the bivariate regressions between all psychosocial variables and pain-related disability, with associations ranging from 0.31 to 0.46 (shown in orange in Fig. 2). These associations remained significant and robust even after adjusting for demographic variables in the bivariate regressions (displayed in yellow in Fig. 2). For detailed results see Tables 3 and 4 and Supplementary Table S1 and S2.

**Table 3:** Bivariate and adjusted associations between the independent variables and pain-related disability.

Variable	Unadjusted					Adjusted						
	B	(SE)	β	p	n	B	(SE)	β	p	n	Part	%
Pain intensity (NRS)	4.010	0.140	0.421	< 0.001	3825	3.429	0.142	0.360	< 0.001	3543	0.348	12.11
Pain bothersomeness (NRS)	3.508	0.149	0.355	< 0.001	3821	3.159	0.147	0.319	< 0.001	3540	0.314	9.86
Pain catastrophizing (PCS)	0.570	0.020	0.423	< 0.001	3720	0.498	0.020	0.369	< 0.001	3443	0.362	13.10
Psych.distress (HSCL-25)	13.244	0.420	0.452	< 0.001	3865	11.942	0.425	0.407	< 0.001	3576	0.393	15.44
Perceived injustice (IEQ)	0.626	0.022	0.417	< 0.001	3731	0.549	0.023	0.364	< 0.001	3455	0.351	12.32
Sleep (ISI)	1.159	0.037	0.458	< 0.001	3757	1.024	0.037	0.405	< 0.001	3476	0.394	15.52
Fatigue (CFQ)	1.755	0.080	0.336	< 0.001	3762	1.662	0.078	0.319	< 0.001	3481	0.315	9.92
Self-efficacy (GSE)	− 9.581	0.475	− 0.313	< 0.001	3758	− 7.360	0.479	− 0.241	< 0.001	3476	− 0.233	5.43

–The adjusted associations were controlled for age, gender, education level and work.

**Table 4:** All independent variables (incl. covariates) in the same regression model (n = 3321).

Dependent variable: pain-related disability (ODI)	B	(SE)	β	p	Part
Model 1 (covariates only)					
Age	0.056	0.019	0.049	0.003	0.047
Gender	10.062	0.560	0.031	0.058	0.030
Education level	− 0.887	0.337	− 0.044	0.009	− 0.042
Work	12.231	0.591	0.353	< 0.001	0.332
Model 2 (the combined model)					
Age	0.102	0.017	0.089	** < 0.001**	0.081
Gender	0.280	0.471	0.008	0.553	0.008
Education level	0.314	0.286	0.015	0.273	0.015
Work	7.419	0.515	0.214	** < 0.001**	0.193
Pain intensity (NRS)	1.811	0.186	**0.193**	** < 0.001**	0.130
Pain bothersomeness (NRS)	0.486	0.193	0.049	0.012	0.034
Pain catastrophe (PCS)	0.038	0.031	0.028	0.219	0.016
Psych.distress (HSCL-25)	3.128	0.660	**0.107**	** < 0.001**	0.063
Perceived injustice (IEQ)	0.100	0.034	0.066	0.003	0.039
Sleep (ISI)	0.493	0.042	**0.196**	** < 0.001**	0.157
Fatigue (CFQ)	0.450	0.086	**0.087**	** < 0.001**	0.070
Self-efficacy (GSE)	− 1.386	0.501	− 0.045	0.006	− 0.037

Significant values are in bold.

**Figure 3:**
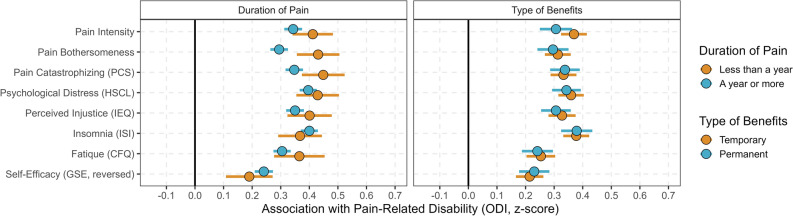
Associations across pain duration and type of benefit between psychosocial variables and pain-related disability adjusted for age, gender, education level and work.

The results from the moderation analyses, conducted using hierarchical regression analyses with interaction terms, revealed only a few significant differences, none of which were large (see Fig. 3). Pain duration played a moderating role for pain bothersomeness (< 0.001) and pain catastrophizing (p = 0.013) on pain-related disability, with slightly stronger associations among individuals who had experienced pain for less than one year than for a year or more. Financial benefits did not moderate any of the associations. For full results, please see Supplementary Table S3 and S4.

When we included all psychosocial variables (including pain intensity) in the multiple regression model, while also being adjusted for gender, age, education, and employment we found that they collectively explained 40.9% (r2 = 0.409) (covariates 14.4%, (r2 = 0.144) and psychosocial variables 26.5%) of the total variance in pain-related disability (F (12,3308) = 191.089, p < 0.000). Most associations were unsurprisingly substantially reduced (shown in blue in Fig. 2). Following an examination for multicollinearity, it was observed that all Variance Inflation Factors (VIFs) remained below 3, representing a conservative threshold. This analysis indicates the absence of significant multicollinearity concerns among the predictor variables in the regression model, thereby negating the necessity for variable removal. Notably, insomnia and pain intensity were the most strongly associated with pain-related disability when adjusting for all the psychosocial factors. For more information, see Table 4.

## Discussion

As hypothesized, we found significant correlations between all the included psychosocial variables we investigated and pain-related disability. These associations remained robust even after adjusting for common demographic factors such as age, gender, education level, and work status. These findings align with previous research, suggesting that psychosocial variables have a central role in pain-related disability.

The correlation between pain intensity and pain-related disability was smaller than anticipated. One might intuitively assume a stronger relationship between pain intensity and disability when a questionnaire specifically addresses pain-related limitations. Typically, as pain intensity increases, the level of disability would also increase. However, the smaller-than-expected correlation implies that additional factors play a significant role in influencing pain-related disability. This underscores the importance of investigating different factors as potential contributors to the complex relationship between pain and disability.

We also found that pain-related disability was about equally correlated with psychological distress as with pain intensity. This relationship may be reciprocal, but the finding aligns with previous research showing that individuals with higher distress levels report elevated pain intensity and pain-related disability^19^. Furthermore, we found a significant correlation between perceived injustice and pain-related disability, which was also at a similar level as pain intensity and pain-related disability. This suggests that this relatively underexplored factor may exert a relevant influence, with the caveat that the causality of this relationship remains uncertain. Perceived injustice encompasses worry or concern, feelings of helplessness, and negative outlooks for the future. This factor is known to correlate with catastrophic thinking about pain^25^. These thought patterns can lead to the monitoring of pain signals, thereby exacerbating the pain experience and contributing to the vicious circle elucidated by the fear-avoidance model. Importantly, our observations regarding the relationship between perceived injustice and pain-related disability are consistent with findings from previous studies^25^. Notably, a recent systematic review reported significant associations between perceived injustice and pain intensity and disability^68^.

Fear, worry and helplessness can trigger physiological responses trough the activation of the sympathetic nervous system, the body’s “fight or flight” response^29^. Chronic stress activation, characterized by feelings of helplessness and hopelessness as opposed to coping, can have profound implications for health, such as cardiovascular issues, immune system dysfunction, and even sleep disturbances, such as insomnia or disrupted sleep patterns, similar to pain-mechanisms in social rejection contexts^27^. Insomnia is often an early sign of persistent stress, but the relationship may be bidirectional. Prolonged stress can lead to poor sleep, while inadequate sleep can trigger stress. In our study, we observed a strong association between sleep problems and pain-related disability, about as strong as for pain-intensity. The association is consistent with previous research showing that individuals with chronic pain often experience sleep disturbances^30^. Furthermore, it’s worth noting that research suggest a potential link between poor sleep and increased pain sensitivity^69^.

The Cognitive Activation Theory of Stress (CATS) also serves as a foundational framework for understanding the relationship between physical activation and fatigue^70^. This state of persistent activation can result in poor sleep patterns, but also trigger automatic and adaptive “alarms”, leading to behavioral changes, such as the emergence of symptoms like pain or fatigue. Chronic stress activation can also lead to psychological effects like worry and rumination, contributing to persistent activation and ultimately fatigue. Notably, since fatigue is common among individuals with chronic pain, with 60–70% reporting co-occurring persistent fatigue^34,71^, the association between fatigue and pain-related disability in our study was expected. Additionally, psychological distress and pain are closely related to fatigue; the longer and more intense pain the more pronounced the fatigue^72^. While fatigue was indeed associated with pain-related disability, the relationship between catastrophizing, psychological distress, perceived injustice, sleep problems and pain-related disability was even stronger. These results are in line with earlier literature but challenge previous perceptions that pain intensity is the primary driver of pain-related disability.

Self-efficacy, defined as the belief in one’s ability to cope with challenges, can positively impact various psychosocial factors and pain-related disability^73^. General self-efficacy, as measured in this study, encompasses aspects of optimism and coping with life in general, rather than specifically focusing on pain-related self-efficacy. It is important to discuss the implications and limitations of using general self-efficacy in a chronic pain population. Few studies have examined general self-efficacy in the context of chronic pain to determine whether it represents domain-specific coping or general coping skills and how it correlates with functional outcomes. Our study identified a negative correlation between self-efficacy and pain-related disability, indicating that low self-efficacy is associated with increased pain-related disability. This observation is in line with previous research suggesting that self-efficacy mediates the impact of pain intensity on pain-related disability^73^.

The observed interaction between pain duration, pain bothersomeness, and pain-related disability is a noteworthy finding. It suggests that as pain persists over time, the perceived bothersomeness of the pain may gradually diminish in its impact on daily functioning. One might speculate that individuals experiencing chronic pain may develop adaptive mechanisms or coping strategies over time, which allow them to better manage and adapt to the presence of persistent pain. These adaptations might reduce the perceived interference of pain with their daily lives. Additionally, it highlights the dynamic nature of pain experiences and the need to consider how pain perceptions and their impact may evolve with longer pain durations.

When evaluating the combined contribution of psychosocial factors to the variation in pain-related disability, we found that these factors accounted for a quarter of the variance. Sleep problems and pain intensity emerged as primary contributors, complemented by psychological distress and fatigue. Notably, the contribution of pain catastrophizing, perceived injustice and self-efficacy were non-significant in the combined regression model, which contrasts with the typical significance of pain catastrophizing in overall regression models. One possible explanation for this discrepancy could be the inclusion of sleep and/or fatigue variables, which may not be as commonly examined. This does not necessarily diminish their importance but may suggest that they may be overshadowed when combined in a multiple regression model. Another possibility is that pain catastrophizing indeed plays a smaller role than other psychosocial variables when it comes to pain-related disability. Supporting this perspective, a systematic review by Lee et al.^41^ revealed that pain catastrophizing did not mediate the link between pain intensity and disability, which contradicts its mediating role in experimental treatment studies^74^. Lee et al. speculate that while catastrophizing may mediate treatment effects, it may be less important for disability development. This intriguing disparity warrants further exploration.

Nevertheless, it is crucial to recognize that the findings from our multiple regression model, while significant, provide just a piece of the full puzzle. Pain-related disability is a multifaced outcome influenced by a range of factors, and our study concentrated on the psychosocial aspects. Many other variables are likely to influence pain-related disability. These include underlying medical conditions, lifestyle factors, cultural influences, and social determinants. The relative scarcity of social variables in our analysis is a particular issue that should be followed up in future studies. Understanding the multifaceted relationship between pain intensity, psychosocial variables, and pain-related disability is still crucial for developing more comprehensive and effective interventions in the field of pain management and rehabilitation.

## Strengths, limitations, and future research

Our study offers several notable strengths. It benefits from a substantial sample size, drawn from a real-world environment at Norway’s largest multidisciplinary pain clinic. The research is underpinned by the application of validated questionnaires and is firmly grounded within a robust theoretical framework. Nevertheless, it’s crucial to acknowledge that the cross-sectional nature of our study design precludes the ability to infer causality between the variables assessed. Additionally, the predominance of psychological over social variables in our analysis represents a limitation. Future investigations should aim to incorporate a broader selection of social variables to more fully elucidate the psychosocial dimensions at play. Regarding treatment implications, there are several opportunities to explore promising interventions. Treatments such as cognitive behavioural therapy for insomnia and Pain Reprocessing Therapy have demonstrated great potential^75,76^ that merits further exploration in pain clinic populations. Furthermore, we advocate for a broader approach to interventions. Comprehensive strategies should not only tackle psychosocial factors but should also encompass a holistic perspective on pain management and disability prevention. Recognizing the significant role of psychosocial factors as modifiable prognostic elements^77^, future research should delve into multifaceted approaches that take into account the interconnected nature of pain intensity, psychological distress, and insomnia.

## Conclusion

In summary, our cross-sectional bi-variate analyses revealed significant associations between pain intensity, pain catastrophizing, psychological distress, perceived injustice, sleep, fatigue, and self-efficacy with pain-related disability, aligning with previous research. In the multiple regression model, sleep problems and pain intensity were identified as primary contributors, alongside psychological distress, and fatigue. Interestingly, pain catastrophizing, perceived injustice, and self-efficacy did not show significant contributions in the combined regression model, which contrasts most previous findings. While the direction of causation remains unclear, these findings emphasize the potential for interventions targeting these psychosocial factors to be more effective in improving the lives of individuals with chronic pain compared to solely focusing on pain management.

### Supplementary Information

Supplementary Tables.

## Data Availability

The data utilized in this study are sourced from the Oslo Pain Registry (OPR). Access to the dataset is granted through the OPR board. Requests to access the data should be directed to chrekh@ous-hf.no.

## Code availability

SPSS version 27.
