# Supplementary material for: Associations between pain intensity, psychosocial factors, and pain-related disability in 4285 patients with chronic pain
Source: Sci Rep. 2024 Jun 12;14:13477. doi: 10.1038/s41598-024-64059-8 (PMC11169509; doi:10.1038/s41598-024-64059-8)
Supplement: Supplementary file 1 — Supplementary Tables. [file 41598_2024_64059_MOESM1_ESM.pdf]

The role of pain intensity and psychosocial factors in how pain impacts daily functioning: A study of 4,285 patients with chronic pain

Live Landmark[1*], Hans Fredrik Sunde[4], Egil A. Fors[2], Leif Edward Ottesen Kennair[1], Annahita Sayadian[3], Caroline Backelin[3], Silje Endresen Reme[5]

[1] Department of Psychology, Faculty of Social and Educational Sciences, Norwegian University of Science and Technology, Trondheim, Norway

[2] Department of Public Health and Nursing, Faculty of Medicine and Health Sciences, Norwegian University of Science and Technology, Trondheim, Norway

[3] Department of Psychology, Faculty of Social Sciences, University of Oslo, Oslo, Norway

[4] Centre for Fertility and Health, Norwegian Institute of Public Health, Oslo, Norway

[5] Department of Pain Management and Research, Oslo University Hospital, Oslo, Norway

Corresponding author: Live Landmark. Email: Livelan@stud.ntnu.no

Supplementary Table S1, Table 3., unadjusted.

Bivariate analyses on the independent variables pain intensity, pain bothersomeness, pain catastrophizing, psychological distress, perceived injustice, self-efficacy, sleep, fatigue, and dependent variable pain-related disability.

Variable	Univariate B	(SE)	β	p	N	F(df1, df2)	p	r2
Pain intensity (NRS)	4.010	.140	.421**	**<.001**	3825	F (1,3823) = 825.861	**<.001**	.178
Pain bothersomeness (NRS)	3.508	.149	.355**	**<.001**	3821	F (1,3819) = 551.618	**<.001**	.126
Pain Catastrophizing (PCS)	.570	.020	.423**	**<.001**	3720	F (1,3718) = 812.315	**<.001**	.179
Psych.distress (HSCL-25)	13.244	.420	.452**	**<.001**	3865	F (1,3863) = 993.620	**<.001**	.205
Perceived injustice (IEQ)	.626	.022	.417**	**<.001**	3731	F (1,3729) = 784.992	**<.001**	.174
Sleep (ISI)	1.159	.037	.458**	**<.001**	3757	F (1,3755) = 999.443	**<.001**	.210
Fatigue (CFQ)	1.755	.080	.336*	**<.001**	3762	F (1,3760) = 478.580	**<.001**	.113
Self-efficacy (GSE)	´-9.581	.475	`-.313*	**<.001**	3758	F (1,3756) = 407.334	**<.001**	.098

Correlation level: *low ≥.15, **moderate ≥.35, *** high ≥.55

Supplementary Table S2, Table 3., adjusted.

Bivariate analyses on the independent variables pain intensity, pain bothersomeness, pain catastrophizing, psychological distress, perceived injustice, sleep, fatigue, self-efficacy, and the dependent variable pain-related disability, controlled for age, gender, education level and work status.

**Variables**	**B**	**(SE)**	**β**	**p**	**N**	**Part**
**Model 1**						
Age	.048	.019	.042	.010		
Gender	.819	.547	.023	.134		
Education level	-.897	.329	-.044	.006		
Work	12.636	.583	**.358**	**<.001**		
**Model 2**						
Age	.009	.017	.008	.587		
Gender	-.058	.508	-.002	.909		
Education level	.145	.308	.007	.638		
Work	10.798	.545	**.306**	**<.001**		
Pain intensity	3.429	.142	**.360**	**<.001**	3543	**.348**

Dependent Variable: Pain-related disability (ODI)

**Variables**	**B**	**(SE)**	**β**	**p**	**N**	**Part**
**Model 1**						
Age	.051	.019	.044	.007		
Gender	.919	.546	.026	.093		
Education level	-.923	.329	-.045	.005		
Work	12.593	.582	**.358**	**<.001**		
**Model 2**						
Age	.077	.018	**.067**	**<.001**		
Gender	.321	.514	.009	.533		
Education level	-.343	.310	-.017	.269		
Work	11.366	.550	**.323**	**<.001**		
Pain bothersomeness	**3.159**	**.147**	**.319**	**<.001**	3540	**.314**

Dependent Variable: Pain-related disability (ODI)

**Variables**	**B**	**(SE)**	**β**	**p**	**N**	**Part**
**Model 1**						
Age	.055	.019	.048	.003		
Gender	1.128	.553	.032	.041		
Education level	-.791	.332	-.039	.017		
Work	12.415	.586	**.355**	**<.001**		
**Model 2**						
Age	.065	.017	**.056**	**<.001**		
Gender	1.204	.509	.035	.018		
Education level	.027	.307	.001	.930		
Work	**10.473**	**.545**	**.300**	**<.001**		
Pain catastrophe (PCS)	**.498**	**.020**	**.369**	**<.001**	3443	**.362**

Dependent Variable: Pain-related disability (ODI)						
**Variables**	**B**	**(SE)**	**β**	**p**	**N**	**Part**
**Model 1**						
Age	.053	.019	.046	.005		
Gender	.945	.544	.027	.083		
Education level	-.910	.327	-.044	.005		
Work	12.578	.580	**.357**	**<.001**		
**Model 2**						
Age	.128	.017	**.111**	**<.001**		
Gender	.099	.493	.003	.841		
Education level	-.510	.296	-.025	.085		
Work	9.371	.537	**.266**	**<.001**		
Psych.distress (HSCL-25)	11.942	.425	**.407**	**<.001**	3576	**.393**

Dependent Variable: Pain-related disability (ODI)						
**Variables**	**B**	**(SE)**	**β**	**p**	**N**	**Part**
**Model 1**						
Age	.050	.019	.043	.008		
Gender	.915	.551	.026	.097		
Education level	-.799	.331	-.039	.016		
Work	12.505	.585	**.358**	**<.001**		
**Model 2**						
Age	.114	.018	**.100**	**<.001**		
Gender	.816	.510	.023	.110		
Education level	-.308	.307	-.015	.315		
Work	9.597	.554	**.275**	**<.001**		
Perceived injustice (IEQ)	.549	.023	**.364**	**<.001**	3455	**.351**

Dependent Variable: Pain-related disability (ODI)						
**Variables**	**B**	**(SE)**	**β**	**p**	**N**	**Part**
**Model 1**						
Age	.050	.019	.044	.008		
Gender	.829	.550	.024	.132		
Education level	-.728	.331	-.036	.028		
Work	12.488	.585	**.357**	**<.001**		
**Model 2**						
Age	.083	.017	**.073**	**<.001**		
Gender	1.479	.498	.043	.003		
Education level	-.343	.300	-.017	.253		
Work	9.527	.540	**.272**	**<.001**		
Sleep (ISI)	1.024	.037	**.405**	**<.001**	3476	**.394**

Dependent Variable: Pain-related disability (ODI)						
**Variables**	**B**	**(SE)**	**β**	**p**	**N**	**Part**
**Model 1**						
Age	.056	.019	.049	.003		

Gender	1.019	.549	.029	.063		
Education level	-.747	.330	-.037	.024		
Work	12.385	.583	.355	<.001		
**Model 2**						
Age	.105	.018	**.092**	**<.001**		
Gender	.506	.517	.015	.328		
Education level	-.954	.310	-.047	.002		
Work	11.000	.552	**.315**	**<.001**		
Fatigue (CFQ)	1.662	.078	**.319**	**<.001**	3481	**.315**
Dependent Variable: Pain-related disability (ODI)						
**Variables**	**B**	**(SE)**	**β**	**p**	**N**	**Part**
**Model 1**						
Age	.056	.019	.049	.003		
Gender	.885	.549	.025	.108		
Education level	-.836	.330	**-.041**	**.011**		
Work	12.368	.584	**.354**	**<.001**		
**Model 2**						
Age	.067	.018	**.059**	**<.001**		
Gender	.800	.532	.023	.133		
Education level	-.296	.321	-.015	.358		
Work	10.589	.577	**.303**	**<.001**		
Self-efficacy (GSE)	-7.360	.479	**-.241**	**<.001**	3476	**-.233**
Dependent Variable: Pain-related disability (ODI)						

Supplementary Table S3.
Moderator analyses with financial disability benefit moderating the relationship between the significant independent variables pain intensity and bothersomeness, pain catastrophizing, psychological distress, perceived injustice, sleep, fatigue and self-efficacy in the adjusted model and the dependent variable pain-related disability.

**Variable**	**B**	**(SE)**	**β**	**t**	**p**	R^2	Adj.R^2
**Model 1**					<.001	.216	.214
(Constant)	-.843	.119		-7.066	<.001		
Age	.001	.002	.018	.886	.376		
Gender	-.066	.035	-.035	-1.863	.063		
Education level	.036	.022	.032	1.668	.096		
Work	.496	.040	.244	12.419	<.001		
Pain intensity	.345	.018	.358	18.993	<.001		
Financial benefit	.021	.038	.011	.534	.593		
**Model 2**					.083	.217	.214
(Constant)	-.852	.119		-7.140	<.001		
Age	.001	.002	.019	.955	.340		
Gender	-.066	.035	-.035	-1.879	.060		
Education level	.036	.022	.032	1.666	.096		
Work	.496	.040	.244	12.426	<.001		
Pain intensity	.432	.054	.448	8.059	<.001		
Financial benefit	.026	.039	.014	.683	.495		
**int_Fin_PainIntens**	-.063	.036	-.097	-1.732	.083		

Dependent variable: Pain-related disability (ODI).

Independent variable: Pain intensity.

Financial benefit= Sick leave/AAP/disability.

Moderator: InterFinPain= Pain Intensity x Financial benefit.

Z-score on all variables except age, gender, education level and work status.

**Variable**	**B**	**(SE)**	**β**	**t**	**p**	R^2	Adj.R^2
**Model 1**					<.001	.196	.194
(Constant)	-1.050	.120		-8.753	<.001		
Age	.005	.002	.071	3.555	<.001		
Gender	-.040	.036	-.021	-1.131	.258		
Education level	.007	.022	.006	.322	.747		
Work	.521	.040	.257	12.956	<.001		
Pain bothersomeness	.306	.018	.322	17.185	<.001		
Financial benefit	.023	.039	.012	.595	.552		
**Model 2**					.639	.196	.194
(Constant)	-1.053	.120		-8.765	<.001		
Age	.005	.002	.072	3.569	<.001		
Gender	-.040	.036	-.021	-1.122	.262		
Education level	.007	.022	.006	.326	.745		
Work	.521	.040	.257	12.948	<.001		
Pain bothersomeness	.330	.054	.347	6.145	<.001		

Financial benefit	.024	.039	.013	.620	.535		
**int_Fin_PainBother**	-.017	.036	-.026	-.470	.639		

Dependent variable: Pain-related disability (ODI).

Independent variable: Pain bothersomeness.

Financial benefit= Sick leave/AAP/disability.

Moderator: InterFinPainBother= Pain Bothersomeness x Financial benefit.

Z-score on all variables except age, gender, education level and work status.

**Variable**	**B**	**(SE)**	**β**	**t**	**p**	R^2	Adj.R^2
**Model 1**					<.001	.219	.217
(Constant)	-1.159	.120		-9.683	<.001		
Age	.005	.002	.060	2.996	.003		
Gender	.008	.035	.004	.235	.815		
Education level	.032	.022	.028	1.473	.141		
Work	.496	.040	.247	12.445	<.001		
Pain catastrophe (PCS)	.335	.017	.360	19.195	<.001		
Financial benefit	.054	.039	.029	1.401	.161		
**Model 2**					.883	.219	.217
(Constant)	-1.158	.120		-9.677	<.001		
Age	.005	.002	.060	2.982	.003		
Gender	.008	.035	.004	.238	.812		
Education level	.032	.022	.028	1.474	.141		
Work	.496	.040	.247	12.441	<.001		
Pain catastrophe (PCS)	.328	.053	.353	6.198	<.001		
Financial benefit	.054	.039	.029	1.394	.164		
**int_Fin_Catastroph**	.005	.035	.008	.147	.883		

Dependent variable: Pain-related disability (ODI).

Independent variable: Pain catastrophizing (PCS).

Financial benefit= Sick leave/AAP/disability.

Moderator: InterFinCatastroph= Catastrophizing (PCS) x Financial benefit.

Z-score on all variables except age, gender, education level and work status.

**Variable**	**B**	**(SE)**	**β**	**t**	**p**	R^2	Adj.R^2
**Model 1**					<.001	.232	.230
(Constant)	-1.041	.116		-8.953	<.001		
Age	.007	.001	.088	4.522	<.001		
Gender	-.045	.034	-.024	-1.296	.195		
Education level	.000	.021	.000	.012	.990		
Work	.448	.039	.222	11.409	<.001		
Psych.distress (HSCL-25)	.352	.017	.379	20.615	<.001		
Financial benefit	.060	.038	.032	1.586	.113		
**Model 2**					.633	.232	.230
(Constant)	-1.043	.116		-8.963	<.001		
Age	.007	.001	.089	4.537	<.001		
Gender	-.045	.034	-.024	-1.306	.192		

Education level	.000	.021	.000	.000	1.000		
Work	.447	.039	.222	11.371	<.001		
Psych.distress (HSCL-25)	.375	.052	.404	7.268	<.001		
Financial benefit	.062	.038	.033	1.631	.103		
**int_Fin_HSCL**	-.016	.034	-.026	-.478	.633		

Dependent variable: Pain-related disability (ODI).

Independent variable: Psychological distress (HSCL-25).

Financial benefit= Sick leave/AAP/disability.

Moderator: InterFinHSCL= Psychological distress (HSCL-25) x Financial benefit.

Z-score on all variables except age, gender, education level and work status.

**Variable**	**B**	**(SE)**	**(β)**	**t**	**p**	**R^2**	**Adj.R^2**
**Model 1**					<.001	.204	.202
(Constant)	-1.141	.120		-9.502	<.001		
Age	.007	.002	.088	4.350	<.001		
Gender	-.005	.036	-.003	-.133	.894		
Education level	.009	.022	.008	.408	.683		
Work	.460	.041	.229	11.339	<.001		
Perceived injustice (IEQ)	.318	.018	.338	17.701	<.001		
Financial benefit	.057	.039	.030	1.451	.147		
**Model 2**					.548	.204	.202
(Constant)	-1.142	.120		-9.510	<.001		
Age	.007	.002	.089	4.380	<.001		
Gender	-.005	.036	-.003	-.148	.882		
Education level	.009	.022	.008	.390	.697		
Work	.458	.041	.228	11.248	<.001		
Perceived injustice (IEQ)	.349	.055	.371	6.376	<.001		
Financial benefit	.059	.039	.032	1.504	.133		
**int_Fin_IEQ**	-.021	.036	-.035	-.600	.548		

Dependent variable: Pain-related disability (ODI).

Independent variable: Injustice (IEQ).

Financial benefit= Sick leave/AAP/disability.

Moderator: InterFinIEQ= Injustice (IEQ) x Financial benefit.

Z-score on all variables except age, gender, education level and work status.

**Variable**	**B**	**(SE)**	**β**	**t**	**p**	**R^2**	**Adj.R^2**
**Model 1**					<.001	.241	.239
(Constant)	-1.057	.117		-9.019	<.001		
Age	.005	.001	.059	3.021	.003		
Gender	.028	.035	.015	.799	.424		
Education level	.016	.021	.014	.760	.447		
Work	.451	.039	.224	11.422	<.001		
Sleep (ISI)	.378	.018	.393	21.171	<.001		
Financial benefit	.020	.038	.011	.515	.606		

**Model 2**					.965	.241	.239
(Constant)	-1.057	.117		-9.008	<.001		
Age	.005	.001	.059	3.020	.003		
Gender	.028	.035	.015	.799	.424		
Education level	.016	.021	.014	.757	.449		
Work	.451	.040	.224	11.395	<.001		
Sleep (ISI)	.376	.054	.390	7.004	<.001		
Financial benefit	.019	.039	.010	.498	.619		
**int_Fin_ISI**	.002	.036	.002	.044	.965		

Dependent variable: Pain-related disability (ODI).

Independent variable: Sleep (ISI).

Financial benefit= Sick leave/AAP/disability.

Moderator: InterFinISI= Sleep (ISI) x Financial benefit.

Z-score on all variables except age, gender, education level and work status.

**Variable**	**B**	**(SE)**	**β**	**t**	**p**	**R^2**	**Adj.R^2**
Model 2					<.001	.157	.155
(Constant)	-1.118	.123		-9.065	<.001		
Age	.005	.002	.070	3.402	<.001		
Gender	-.035	.036	-.019	-.962	.336		
Education level	-.019	.022	-.017	-.853	.394		
Work	.537	.041	.267	13.052	<.001		
Fatigue (CFQ)	.248	.019	.254	13.188	<.001		
Financial benefit	.072	.040	.039	1.807	.071		
**Model 2**					.747	.157	.154
(Constant)	-1.119	.123		-9.069	<.001		
Age	.005	.002	.070	3.389	<.001		
Gender	-.035	.037	-.019	-.968	.333		
Education level	-.019	.022	-.017	-.851	.395		
Work	.537	.041	.267	13.032	<.001		
Fatigue (CFQ)	.266	.058	.273	4.576	<.001		
Financial benefit	.074	.040	.040	1.834	.067		
**int_Fin_CFQ**	-.012	.038	-.019	-.323	.747		

Dependent variable: Pain related disability.

Independent variable: Fatigue (CFQ).

Financial benefit= Sick leave/AAP/disability.

Moderator: InterFinCFQ= Fatigue (CFQ) x Financial benefit.

Z-score on all variables except age, gender, education level and work status.

**Variable**	**B**	**(SE)**	**β**	**t**	**p**	**R^2**	**Adj.R^2**
**Model 1**					<.001	.148	.145
(Constant)	-1.081	.124		-8.723	<.001		
Age	.005	.002	.063	3.010	.003		
Gender	-.010	.037	-.005	-.262	.793		

Education level	.013	.023	.011	.565	.572		
Work	.496	.042	.246	11.865	<.001		
Self-efficacy (GSE)	-.222	.018	-.238	-12.103	<.001		
Financial benefit	.042	.040	.023	1.049	.294		
**Model 2**					.658	.148	.145
(Constant)	-1.079	.124		-8.709	<.001		
Age	.005	.002	.063	2.996	.003		
Gender	-.009	.037	-.005	-.253	.801		
Education level	.013	.023	.011	.562	.574		
Work	.497	.042	.247	11.868	<.001		
Self-efficacy (GSE)	-.198	.056	-.213	-3.568	<.001		
Financial benefit	.040	.041	.022	.995	.320		
**int_Fin_GSE**	-.016	.036	-.026	-.443	.658		

Dependent variable: Pain-related disability (ODI).

Independent variable: Self-efficacy (GSE).

Financial benefit= Sick leave/AAP/disability.

Moderator: InterFinGSE= Self-efficacy x Financial benefit.

Z-score on all variables except age, gender, education level and work status.

Supplementary Table S4.
Moderator analyses with duration of pain moderating the relationship between the significant independent variables pain intensity, bothersomeness, pain catastrophizing, psychological distress, perceived injustice, sleep, fatigue and self-efficacy in the adjusted model and the dependent variable pain-related disability.

**Variable**	**B**	**(SE)**	**β**	**t**	**p**	**R^2**	**Adj.R^2**
**Model 1**					<.001	.268	.266
(Constant)	-1.035	.122		-8.476	<.001		
Age	.000	.001	.007	.490	.624		
Gender	-.004	.029	-.002	-.144	.885		
Education level	.009	.018	.008	.504	.614		
Work	.620	.031	.307	19.814	<.001		
Pain intensity	.354	.015	.360	24.171	<.001		
Duration of pain	-.037	.044	-.012	-.842	.400		
**Model 2**					.079	.268	.267
(Constant)	-1.030	.122		-8.440	<.001		
Age	.000	.001	.007	.490	.624		
Gender	-.005	.029	-.002	-.162	.871		
Education level	.009	.018	.008	.525	.600		
Work	.621	.031	.308	19.854	<.001		
Pain intensity	.481	.073	.489	6.548	<.001		
Duration of pain	-.040	.044	-.013	-.914	.361		
**int_Durat_PainIntens**	-.069	.039	-.131	-1.756	.079		

Dependent variable: Pain-related disability (ODI).

Independent variable: Pain intensity.

Duration of pain= 1 year or less / more than 1 year.

Moderator: InterDuratPain= Pain Intensity x Duration of pain.

Z-score on all variables except age, gender, education level and work status.

**Variable**	**B**	**(SE)**	**β**	**t**	**p**	**R^2**	**Adj.R^2**
**Model 1**					<.001	.248	.247
(Constant)	-1.270	.123		-10.290	<.001		
Age	.004	.001	.066	4.340	<.001		
Gender	.018	.029	.009	.605	.545		
Education level	-.019	.018	-.016	-1.083	.279		
Work	.652	.032	.324	20.652	<.001		
Pain bothersomeness	.314	.015	.319	21.532	<.001		
Duration of pain	-.023	.045	-.008	-.523	.601		
**Model 2**					**<.001**	.248	.247
(Constant)	-1.265	.123		-10.269	<.001		
Age	.004	.001	.067	4.374	<.001		
Gender	.016	.029	.008	.560	.575		
Education level	-.018	.018	-.015	-1.006	.314		
Work	.654	.032	.325	20.742	<.001		
Pain bothersomeness	.567	.077	.576	7.335	<.001		
Duration of pain	-.028	.045	-.009	-.634	.526		

**int_Durat_PainBother**	-.136	.041	-.262	-3.334	**<.001**		

Dependent variable: Pain-related disability (ODI).

Independent variable: Pain bothersomeness.

Duration of pain= 1 year or less / more than 1 year.

Moderator: InterFinPainBother= Pain Bothersomeness x Duration of pain.

Z-score on all variables except age, gender, education level and work status.

**Variable**	**B**	**(SE)**	**β**	**t**	**p**	**R^2**	**Adj.R^2**
**Model 1**					<.001	.276	.274
(Constant)	-1.359	.122		-11.155	<.001		
Age	.004	.001	.057	3.733	<.001		
Gender	.069	.029	.035	2.369	.018		
Education level	.001	.018	.001	.080	.936		
Work	.599	.031	.299	19.151	<.001		
Pain catastrophe (PCS)	.362	.015	.369	24.904	<.001		
Duration of pain	.010	.044	.003	.236	.814		
**Model 2**					**.013**	.277	.275
(Constant)	-1.354	.122		-11.121	<.001		
Age	.004	.001	.056	3.701	<.001		
Gender	.067	.029	.033	2.285	.022		
Education level	.001	.018	.001	.038	.969		
Work	.599	.031	.299	19.147	<.001		
Pain catastrophe (PCS)	.550	.078	.562	7.086	<.001		
Duration of pain	.012	.044	.004	.271	.786		
**int_Durat_Catastroph**	-.101	.041	-.196	-2.472	**.013**		

Dependent variable: Pain-related disability (ODI).

Independent variable: Pain catastrophizing (PCS).

Duration of pain= 1 year or less / more than 1 year.

Moderator: InterDuratCatastroph= Catastrophizing (PCS) x Duration of pain.

Z-score on all variables except age, gender, education level and work status.

**Variable**	**B**	**(SE)**	**β**	**t**	**p**	**R^2**	**Adj.R^2**
**Model 1**					<.001	.301	.300
(Constant)	-1.219	.117		-10.376	<.001		
Age	.007	.001	.111	7.499	<.001		
Gender	.006	.028	.003	.199	.842		
Education level	-.029	.017	-.025	-1.718	.086		
Work	.537	.031	.266	17.405	<.001		
Psych.distress (HSCL-25)	.401	.014	.407	28.065	<.001		
Duration of pain	-.003	.042	-.001	-.061	.952		
**Model 2**					.417	.301	.300
(Constant)	-1.216	.117		-10.351	<.001		
Age	.007	.001	.111	7.508	<.001		
Gender	.005	.028	.002	.175	.861		
Education level	-.030	.017	-.025	-1.754	.079		

Work	.536	.031	.266	17.374	<.001		
Psych.distress (HSCL-25)	.462	.077	.470	5.994	<.001		
Duration of pain	-.002	.042	-.001	-.051	.959		
**int_Durat_HSCL**	-.033	.041	-.064	-.812	.417		

Dependent variable: Pain-related disability (ODI).

Independent variable: Psychological distress (HSCL-25).

Duration of pain= 1 year or less / more than 1 year.

Moderator: InterDuratHSCL= Psychological distress (HSCL-25) x Duration of pain.

Z-score on all variables except age, gender, education level and work status.

**Variable**	**B**	**(SE)**	**β**	**t**	**p**	**R^2**	**Adj.R^2**
**Model 1**					<.001	.269	.268
(Constant)	-1.288	.123		-10.503	<.001		
Age	.007	.001	.099	6.467	<.001		
Gender	.046	.029	.023	1.592	.111		
Education level	-.017	.018	-.015	-.995	.320		
Work	.550	.032	.275	17.296	<.001		
Perceived injustice (IEQ)	.357	.015	.364	24.112	<.001		
Duration of pain	-.012	.045	-.004	-.261	.794		
**Model 2**					.228	.269	.268
(Constant)	-1.275	.123		-10.354	<.001		
Age	.007	.001	.099	6.444	<.001		
Gender	.045	.029	.023	1.546	.122		
Education level	-.018	.018	-.015	-1.020	.308		
Work	.549	.032	.275	17.276	<.001		
Perceived injustice (IEQ)	.452	.080	.461	5.632	<.001		
Duration of pain	-.016	.045	-.005	-.349	.727		
**int_Durat_IEQ**	-.051	.042	-.099	-1.206	.228		

Dependent variable: Pain-related disability (ODI).

Independent variable: Perceived injustice (IEQ).

Duration of pain= 1 year or less / more than 1 year.

Moderator: InterDuratIEQ= Perceived injustice (IEQ) x Duration of pain.

Z-score on all variables except age, gender, education level and work status.

**Variable**	**B**	**(SE)**	**β**	**t**	**p**	**R^2**	**Adj.R^2**
**Model 1**					<.001	.300	.298
(Constant)	-1.196	.120		-9.979	<.001		
Age	.005	.001	.072	4.820	<.001		
Gender	.084	.029	.042	2.944	.003		
Education level	-.019	.017	-.016	-1.107	.268		
Work	.547	.031	.273	17.685	<.001		
Sleep (ISI)	.396	.014	.405	27.765	<.001		
Duration of pain	-.044	.044	-.014	-1.015	.310		
**Model 2**					.434	.300	.298
(Constant)	-1.204	.120		-10.009	<.001		

Age	.005	.001	.071	4.784	<.001		
Gender	.084	.029	.042	2.956	.003		
Education level	-.019	.017	-.016	-1.094	.274		
Work	.547	.031	.273	17.682	<.001		
Sleep (ISI)	.335	.079	.343	4.226	<.001		
Duration of pain	-.040	.044	-.013	-.917	.359		
**int_Durat_ISI**	.033	.042	.063	.783	.434		

Dependent variable: Pain-related disability (ODI).

Independent variable: Sleep (ISI).

Duration of pain= 1 year or less / more than 1 year.

Moderator: InterDuratISI= Sleep (ISI) x Duration of pain.

Z-score on all variables except age, gender, education level and work status.

**Variable**	**B**	**(SE)**	**β**	**t**	**p**	**R^2**	**Adj.R^2**
**Modell 1**					<.001	.243	.242
(Constant)	-1.379	.124		-11.144	<.001		
Age	.006	.001	.093	5.950	<.001		
Gender	.030	.030	.015	1.001	.317		
Education level	-.055	.018	-.047	-3.109	.002		
Work	.628	.032	.314	19.800	<.001		
Fatigue (CFQ)	.311	.015	.319	21.333	<.001		
Duration of pain	.043	.045	.014	.942	.346		
**Modell 2**					.202	.244	.242
(Constant)	-1.385	.124		-11.185	<.001		
Age	.006	.001	.093	5.960	<.001		
Gender	.029	.030	.015	.982	.326		
Education level	-.056	.018	-.048	-3.160	.002		
Work	.628	.032	.314	19.803	<.001		
Fatigue (CFQ)	.426	.091	.438	4.665	<.001		
Duration of pain	.047	.045	.015	1.034	.301		
**int_Durat_CFQ**	-.061	.047	-.119	-1.275	.202		

Dependent variable: Pain-related disability (ODI).

Independent variable: Fatigue (CFQ).

Duration of pain= 1 year or less / more than 1 year.

Moderator: InterDuratCFQ= Fatigue (CFQ) x Duration of pain.

Z-score on all variables except age, gender, education level and work status.

**Variable**	**B**	**(SE)**	**β**	**t**	**p**	**R^2**	**Adj.R^2**
**Model 1**					<.001	.199	.197
(Constant)	-1.262	.128		-9.843	<.001		
Age	.004	.001	.059	3.681	<.001		
Gender	.046	.030	.023	1.498	.134		
Education level	-.017	.018	-.014	-.913	.361		
Work	.607	.033	.303	18.323	<.001		
Self-efficacy (GSE)	-.234	.015	-.241	-15.350	<.001		

Duration of pain	-.008	.047	-.003	-.165	.869		
**Model 2**					.252	.199	.198
(Constant)	-1.268	.128		-9.885	<.001		
Age	.004	.001	.059	3.727	<.001		
Gender	.046	.030	.023	1.509	.131		
Education level	-.017	.018	-.014	-.903	.367		
Work	.606	.033	.303	18.317	<.001		
Self-efficacy (GSE)	-.139	.084	-.143	-1.647	.100		
Duration of pain	-.006	.047	-.002	-.127	.899		
**int_Durat_GSE**	-.051	.044	-.100	-1.147	.252		

Dependent variable: Pain-related disability (ODI).

Independent variable: Self-efficacy (GSE).

Duration of pain= 1 year or less / more than 1 year.

Moderator: InterDuratGSE= Self-efficacy (GSE) x Duration of pain.

Z-score on all variables except age, gender, education level and work status.
